# Deep Brain Stimulation for Refractory Obsessive-Compulsive Disorder: Towards an Individualized Approach

**DOI:** 10.3389/fpsyt.2019.00905

**Published:** 2019-12-13

**Authors:** Suhan Senova, Anne-Hélène Clair, Stéphane Palfi, Jérôme Yelnik, Philippe Domenech, Luc Mallet

**Affiliations:** ^1^ AP-HP, Groupe Hospitalier Henri-Mondor, DHU PePsy, Neurosurgery, Psychiatry and Addictology departments, Créteil, France; ^2^ Université Paris Est Creteil, Faculté de Médecine, Créteil, France; ^3^ IMRB UPEC/INSERM U 955 Team 14, Créteil, France; ^4^ Sorbonne Universités, UPMC Univ Paris 06, CNRS, INSERM, Institut du Cerveau et de la Moelle épinière, Paris, France; ^5^ Department of Mental Health and Psychiatry, Global Health Institute, University of Geneva, Geneva, Switzerland

**Keywords:** deep brain stimulation, obsessive compulsive disorder, connectivity, anterior limb of internal capsule, subthalamic nucleus, ventral capsule, ventral striatum, inferior thalamic peduncle

## Abstract

Obsessive-compulsive disorder (OCD) is a neuropsychiatric disorder featuring repetitive intrusive thoughts and behaviors associated with a significant handicap. Of patients, 20% are refractory to medication and cognitive behavioral therapy. Refractory OCD is associated with suicidal behavior and significant degradation of social and professional functioning, with high health costs. Deep brain stimulation (DBS) has been proposed as a reversible and controllable method to treat refractory patients, with meta-analyses showing 60% response rate following DBS, whatever the target: anterior limb of the internal capsule (ALIC), ventral capsule/ventral striatum (VC/VS), nucleus accumbens (NAcc), anteromedial subthalamic nucleus (amSTN), or inferior thalamic peduncle (ITP). But how do we choose the “best” target? Functional neuroimaging studies have shown that ALIC-DBS requires the modulation of the fiber tract within the ventral ALIC *via* the ventral striatum, bordering the bed nucleus of the stria terminalis and connecting the medial prefrontal cortex with the thalamus to be successful. VC/VS effective sites of stimulation were found within the VC and primarily connected to the medial orbitofrontal cortex (OFC) dorsomedial thalamus, amygdala, and the habenula. NAcc-DBS has been found to reduce OCD symptoms by decreasing excessive fronto-striatal connectivity between NAcc and the lateral and medial prefrontal cortex. The amSTN effective stimulation sites are located at the inferior medial border of the STN, primarily connected to lateral OFC, dorsal anterior cingulate, and dorsolateral prefrontal cortex. Finally, ITP-DBS recruits a bidirectional fiber pathway between the OFC and the thalamus. Thus, these functional connectivity studies show that the various DBS targets lie within the same diseased neural network. They share similar efficacy profiles on OCD symptoms as estimated on the Y-BOCS, the amSTN being the target supported by the strongest evidence in the literature. VC/VS-DBS, amSTN-DBS, and ALIC-DBS were also found to improve mood, behavioral adaptability and potentially both, respectively. Because OCD is such a heterogeneous disease with many different symptom dimensions, the ultimate aim should be to find the most appropriate DBS target for a given refractory patient. This quest will benefit from further investigation and understanding of the individual functional connectivity of OCD patients.

## Introduction

Obsessive-compulsive disorder (OCD) affects 2–3% of the general population and is characterized by repetitive, stereotyped, and intrusive thoughts (obsessions) and behaviors (compulsions), leading to a significant disability (1, 2). Cognitive behavioral therapy and medication with selective serotonin reuptake inhibitors (SSRIs) are the two first-line treatments currently recommended for OCD (3). However, 40–60% of patients experience persistent symptoms despite these treatments (2). About 20% of OCD patients are considered to be refractory (4, 5). Even if there is no consensual definition of refractory OCD, levels of non-response have been proposed (6). Treatment-refractory OCD is associated with high comorbidities (including major depression), suicidal behaviors, and severe alterations in social, familial, and professional functioning (leading to significant costs for society) (5).

Current therapeutic options for severe and treatment-resistant OCD include neuromodulation by high-frequency deep brain stimulation (DBS), which is a reversible neurosurgical technique which can be modified and adapted over time and targeted at different nuclei within the fronto-striato-thalamo-cortical network (2). Recent meta-analyses estimate that 60% of treatment-refractory OCD patients respond well to DBS (>35% of decrease on the Y-BOCS severity scale), irrespective of the chosen target (7, 8).

A network, including cognitive and limbic territories of basal ganglia nuclei [ventral striatum (VS) (9), nucleus accumbens (NAcc) (10), and anteromedial subthalamic nucleus (amSTN) (11, 12)] and white matter bundles [anterior limb of the internal capsule (ALIC) (13), ventral capsule (VC), and inferior thalamic peduncle (ITP) (14, 15)] connecting frontal areas to basal ganglia has been proposed to be at the core of OCD physiopathology (16). Such a fronto-striato-thalamo-cortical network has been found to be altered in neuroimaging (17) and anatomical connectivity studies in OCD (18). A precise understanding of functional connectivity of the patients undergoing DBS and of the DBS sites before or after implantation is crucial since DBS should be considered to be a technique to modulate circuits rather than a mere focal target. Its effects are generally considered to be both local *via* somato-dendritic stimulation and long-range *via* orthodromic and antidromic axonal stimulation (19).

Anatomical connectivity can be determined *in vivo* using diffusion-weighted imaging (DWI) acquired by magnetic resonance imaging followed by tractography reconstructions according to deterministic or probabilistic algorithms. It provides streamlines between determined regions, which are hypothetical fibers, estimating the trajectory and the density of the white matter projections between anatomical regions. But the validity of these estimates compared with anatomical reality is a matter of debate (20). Functional connectivity can be determined *in vivo* using resting-state functional MRI, which measures correlation of spontaneous activity between several brain regions (21). Such techniques have recently been used in the field of DBS to guide neurosurgical procedure of DBS electrode implantation (22).

Indeed, despite the numerous advances leading to the current DBS technique, some patients remain partially resistant to DBS. For OCD, recent debates have focused on identifying the "best" DBS target without any major adverse event. We review here and analyze the various DBS targets used to treat refractory OCD patients and highlight how determination of functional connectivity profiles of each patient might be crucial to determine which target might enable modulation of circuits of interest for a given patient. Indeed, the clinical heterogeneity of OCD (23, 24), linked to distinct neural correlates as witnessed by functional neuroimaging (25), and by anatomical variations revealed by connectivity analyses (26) encourage us to define a more personalized target, taking into account the different patterns of impaired neurocognitive processes across OCD patients.

## The Fronto-Striato-Pallido-Thalamic Circuit

Understanding connectivity profiles of the various DBS targets for OCD requires basic knowledge of the prefrontal cortex (PFC) in humans and the fronto-striato-pallido-thalamic circuit. The PFC is associated with central executive functions. It is involved in selecting relevant information and ignoring irrelevant information to drive goal-directed behaviors. Human PFC can be divided into two functionally and anatomically different regions: the medial (MPFC) and the lateral prefrontal cortex (LPFC) (27, 28). The MPFC receives projections from sensory cortices, the hippocampus and subiculum, the amygdala (29–31). It is also connected with the NAcc, the posterior cingulate cortex, the insula, and the hypothalamus (30, 32, 33). As a hub, it integrates information within large-scale brain networks, such as the default-mode network (DMN) and the salience network (SN) (34, 35). The LPFC includes the dorsolateral prefrontal cortex (DLPFC), the ventrolateral prefrontal cortex (VLPFC), and the frontal eye field. The LPFC is involved in higher cognitive functions. The DLPFC, especially, is crucial for the planning and execution of complex temporal sequences of logical reasoning, speech, and behavior supporting both short-term memory, preparatory set, and selective attention (36). The LPFC receives a wealth of information from a large variety of cortical and subcortical structures and in turn projects to the same structures, but distinct areas in the LPFC receive and issue topographically organized projections. More specifically, the DLPFC is a crucial node in dorsal attention networks through its connections with parietal cortex, thus supporting selection of sensory information and response (37).

**Figure 1:**
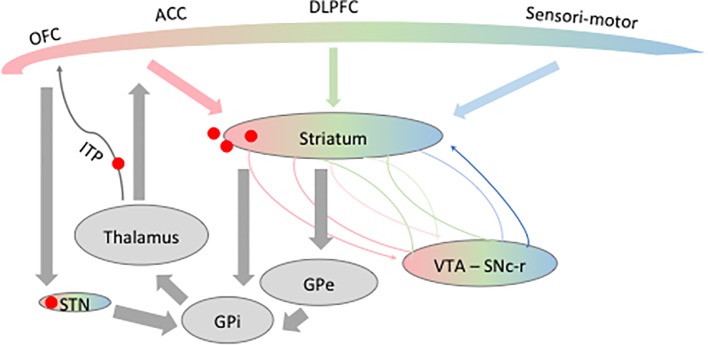
Schematic representation of the fronto-striato-thalamo-cortical circuit. Different frontal areas known to be involved in obsessive-compulsive disorder (OCD) physiopathology are represented here: the orbitofrontal cortex (OFC), anterior cingulate cortex (ACC), and dorsolateral prefrontal cortex (DLPFC). The frontal lobe is composed of limbic (*pink*), associative (*green*), and sensorimotor (*blue*) territories projecting on partially overlapping part of the striatum. The same functional mapping has been shown for the ventral tegmental area (VTA), substantia nigra pars compacta–reticulata (SNc-r), and the subthalamic nucleus (STN). *Red spots* represent the different targets of DBS in OCD discussed in this review. *GPe*, globus pallidus externalis; *GPi*, globus pallidus internalis.

In healthy fronto-striato-thalamo-cortical circuits (**Figure 1**), glutamatergic output from the orbitofrontal cortex (OFC) and the anterior cingulate cortex (ACC) leads to striatal excitation (38–40). Striatal activation through the direct pathway increases inhibitory GABA signals to the globus pallidus internalis (GPi) and the substantia nigra reticulata (SNr). It decreases the inhibitory GABA output from the GPi and SNr to the thalamus, thus enhancing excitatory glutamatergic output from the thalamus to the frontal cortex. This positive-feedback direct pathway is retrocontrolled by the so-called trans-striatal indirect pathway: the striatum inhibits the globus pallidus externalis (GPe) and decreases its inhibition of the subthalamic nucleus (STN) through the indirect pathway. The STN then excites the GPi and SNr, which inhibit the thalamus, and also receives projections directly from the cortex forming a third pathway, the so-called hyperdirect pathway (41).

In OCD patients, it was proposed that the direct pathway is not sufficiently retrocontrolled by the indirect pathway (16). With lower signals of activation, hyperactivation of the orbitofrontal–subcortical pathway appears. Thus, excessive concerns about hygiene or danger might lead to persistent attention to the supposed threat (i.e., obsessions) and then to compulsions, which are phenomenologically associated with the neutralization of the putative threat.

Furthermore, it has been shown that the striatum, as other nuclei of the basal ganglia, is made of three functionally and anatomically distinct territories: motor, limbic, and associative (42–46). How these networks interact with each other is of utmost importance for motivation and cognition to influence decision-making and adaptive behavior. It has been shown that the mesolimbic (through the ventral tegmental area, VTA) and nigrostriatal pathways are an integral part of the basal ganglia through their reciprocal connections to the ventral and dorsal striatum, respectively (47, 48). These mesolimbic loops enable the flow of striatal information from limbic to cognitive to motor circuits through an ascending spiral of inputs and outputs between the striatum and midbrain dopaminergic neurons (47) (**Figure 1**).

The modulation of the fronto-striato-pallido-thalamic circuit of OCD patients has been tested with repeated transcranial magnetic stimulation (rTMS) of different cortical areas (49). Connectivity of the various proposed rTMS targets for OCD could be compared with the connectivity of DBS targets for OCD. Indeed, most rTMS studies targeted the DLPFC (50, 51), the supplementary motor area (SMA) (52–54), and the OFC (55, 56) of OCD patients. The results of these studies have been inconsistent for the DLPFC [(57, 58) vs. (59, 60)], the SMA or OFC. Replication trials following high-quality methodology will help clarify the therapeutic potential of these last two targets. Even though, these three main targets for rTMS seem to share relevant connectivity with the DBS targets for OCD, and amSTN especially (61–63).

## Striatal Region

### Anterior Limb of the Internal Capsule

Within the ventral striatal region, the ALIC was the first target to be explored since, originally, ALIC stereotaxic lesions were performed for OCD: ALIC-DBS led to a significant improvement in three out of four refractory OCD patients (13). Case reports and open studies have reported that about 50% of OCD patients respond favorably to ALIC-DBS (64–68). A recent open-label study, including the largest cohort of 20 refractory OCD patients stimulated in the ALIC, reported 40% of responders 1 year after surgery (68). Adverse events were limited to one case due to hardware infection, and transient effects due to DBS settings, such as hypomania, disinhibition, lack of concentration, transient loss of energy, sleep disturbances, and >20% weight gain (68). If the clinical effects of ALIC-DBS on refractory OCD were encouraging, a confirmation level I study is still needed (69). The identification of clinical and anatomical response predictors to DBS could contribute to a refinement of the ALIC target, personalized targeting, better outcomes, and make such level I studies stronger.

**Figure 2:**
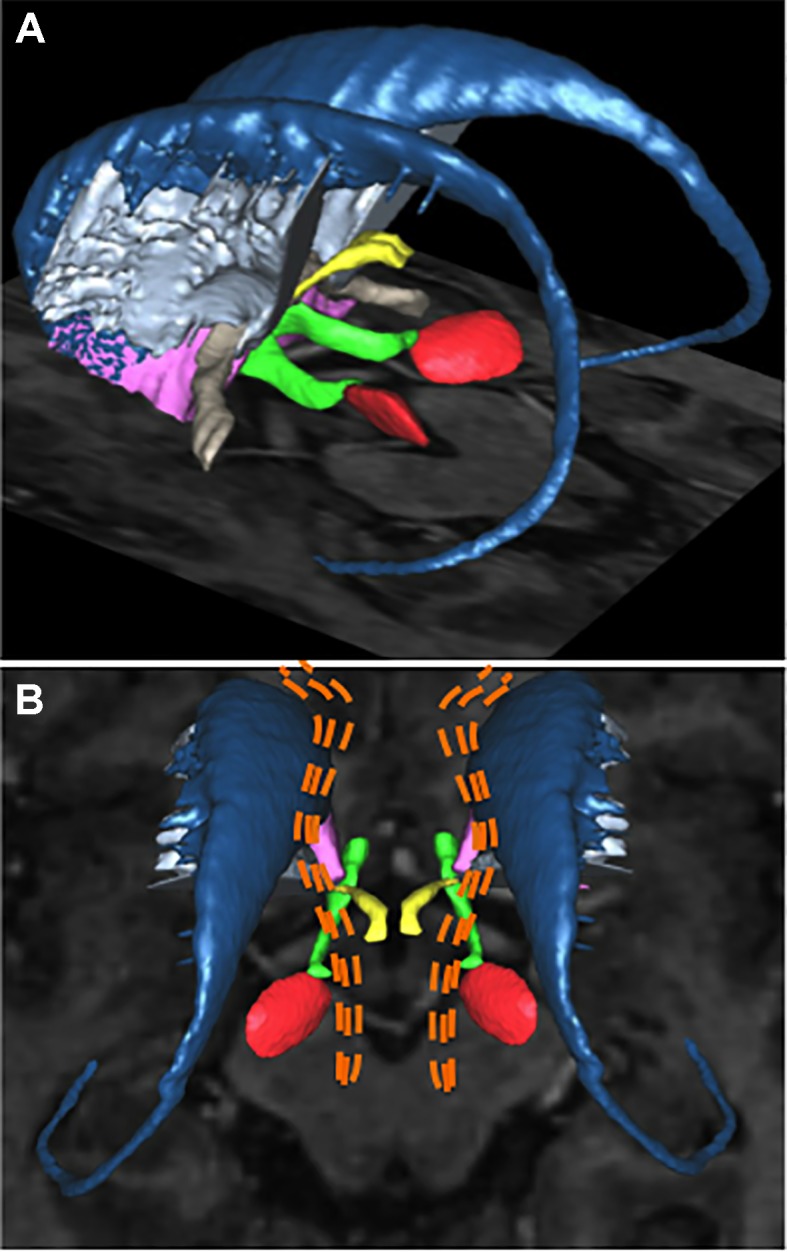
Deep brain stimulation targets for patients with refractory obsessive-compulsive disorder. Basal ganglia structures and adjacent fiber bundles as depicted on 3D reconstructions obtained with the computerized YeB atlas (76). **(A)** Left lateral view showing the structures of both sides. Some of the main DBS targets for OCD are: the subthalamic nucleus (*red*), the anterior limb of internal capsule (*light blue*), the nucleus accumbens (*purple*), and the inferior thalamic peduncle (*yellow*). The caudate nucleus (*dark blue*), medial forebrain bundle (*green*), and anterior commissure (*light brown*) are also shown. **(B)** Superior view showing a schematic representation of hypothetical fibers identified by neuroimaging studies as responsible for the therapeutic effect of DBS for OCD: the fibers are shown as *orange dotted lines*. These fibers, which extend from the brain stem to the prefrontal cortex, encompass the atlas-defined medial forebrain bundle. *DBS*, deep brain stimulation; *OCD*, obsessive-compulsive disorder.

Recently, individual clinical features of OCD were not found to be predictors of response to DBS in a large cohort (*n* = 20) (68). Neuroimaging techniques such as resting-state functional connectivity or DWI have shed a new light on the relationship between anatomical locations of the DBS target and response to stimulation. Using DWI followed by tractography reconstructions in six OCD patients undergoing ALIC-DBS, Hartmann et al. (70) showed that the two best responder target locations within ALIC had a stronger connectivity with the right middle frontal gyrus (MFG), whereas for two non-responders the DBS site had higher connectivity with the right thalamus and the orbital part of the right inferior frontal gyrus. The right MFG is known to be associated with executive functions and adapting sets in response to changing task requests (71), whereas the orbital part of the right inferior frontal gyrus is involved in task-switching and maintenance of compulsive behavior (72–74), so that successful ALIC-DBS might rely at least partially on promoting adaptive responses, but this not yet been studied. More recently, a study using DWI and tractography of anatomical connectivity of ALIC-DBS sites for 22 OCD patients (75) confirmed that a predictor of clinical improvement was the connectivity between stimulation sites within ALIC and the right MFG. More precisely, and as direct guidance for neurosurgeons targeting ALIC, this study highlighted the importance of modulating a fiber tract within the ventral ALIC passing through the ventral striatum, bordering the bed nucleus of the stria terminalis (BNST), the subthalamic nucleus, the inferior thalamic peduncle, and connecting overall the middle prefrontal cortex with the thalamus (**Figure 2**) (76). This result suggests that the different DBS targets reviewed in this manuscript might lie within the same neural network, the modulation of which alleviates OCD core symptoms. On the contrary, modulation of fibers projecting to the medial forebrain bundle, the posterior limb of the anterior commissure, and within the inferior lateral fascicle led to worse outcomes on OCD symptoms. Furthermore, depressive scores were improved when modulating fibers encompassing the cingulum, ventromedial prefrontal cortex, and the fornix. Thus, ALIC-DBS could improve adaptability and/or mood depending on the various fiber pathways affected by DBS after electrical field diffusion at the vicinity of the active DBS site, but this hypothesis remains to be investigated. When performing ALIC-DBS, and after acquiring the anatomical connectivity profile for a given patient, it might be possible to fine-tune and personalize the stimulation parameters in order to address mood or flexibility impairments in addition to OCD core symptoms depending on each patient’s specific symptoms. Last but not least, the high stimulation amplitudes used (median of 4.7 V) in these studies can be questioned: do they simply indicate that the target to be stimulated has a larger volume than the portion of the STN to stimulate in Parkinson’s disease (PD) patients for instance, or are they due to suboptimal electrode placement?

ALIC seems a promising DBS target to treat refractory OCD. ALIC-DBS ability to promote adaptability and/or mood depending on the connectivity of the stimulation site remains to be established. But this DBS target for OCD is lacking confirmation by level I studies, and ALIC optimal targeting seems challenging since it might require precise mapping of ALIC-DBS sites with whole brain anatomical connectivity. This neuroimaging procedure might not be available on a clinical routine basis in all neurosurgical centres and therefore prevent its widespread adoption.

### Ventral Capsule/Ventral Striatum

DBS of the ventral capsule and adjacent ventral striatum (VC/VS) has been investigated in refractory OCD based on the earlier encouraging results following DBS of the ALIC (13) and considering that an additional lesion of the ventral part of the ALIC enhanced the clinical benefit (77). However, the only randomized study available failed to show a significant difference between patients under active (“on”) versus inactive (“off”) stimulation (9). The short duration of the "on" phase in the randomized part of the study, i.e., 2 months only, could explain these negative results. Interestingly, four out of the six OCD patients included were responders after a 1-year open-label phase of DBS. Furthermore, a recent randomized study comparing STN- to VC/VS-DBS in OCD for 12-week phases found that six out of six patients responded to VC/VS-DBS (61). Open studies also showed an improvement of OCD symptoms after VC/VS-DBS over periods longer than 2 months of stimulation (67, 77–80). Most of these studies with up to a 1-year follow-up reached response rates around 50%. Four of four patients were considered as responders in a recent study with a follow-up of 2 years after surgery (79). The follow-up study of six patients previously included in the cohort of Goodman (9) found that 67% of the patients were responders 6 years after surgery (80). In these studies, patients described an increase of OCD symptom severity when the stimulator batteries were depleted (67, 78). Taken together, these results support the efficacy of VC/VS-DBS for patients suffering from refractory OCD with a response rate increasing over time. But no level I study with a long enough phase of active DBS has actually confirmed this potential effect (8). Overall, VC/VS-DBS could be considered as a reasonably safe surgical procedure. All but one patient of the Greenberg cohort (67) indicated that they would choose to have DBS again (80).

VC/VS-DBS was associated with transient cognitive effects: transient hypomania, possibly due a temporary electrode lesioning effect, stimulation-induced memory experiences, and a lasting major decrease of comorbid depressive symptoms (9, 67, 78, 79), suggesting a modulation of the reward and motivational system. Preoperative predictors of clinical response to VC/VS-DBS remain to be determined in larger studies. But a recent study suggested that the VC/VS effective sites of DBS lay within the VC (ventral portion of the ALIC) and were primarily connected to medial OFC, dorsomedial thalamus, amygdala, and the habenula (61). In this study, VC/VS specifically improved the mood of patients. Previously, abnormal functional connectivity assessed by functional MRI (fMRI) was found in OCD patients between the amygdala and medial OFC (81) and medial OFC was found to be hyperresponsive to threat stimuli in OCD patients (82).

So, VC/VS-DBS might both improve OCD core symptoms and mood and/or impair safety signaling depending on the fiber pathways recruited in the vicinity of the active DBS stimulation site. Moreover, it is interesting to note that the habeno-interpeduncular tract is known to inhibit the serotonergic raphe nuclei (83) while SSRIs are the mainstay of pharmacological treatment for OCD.

### Nucleus Accumbens

Several studies have investigated NAcc-DBS: the distinction between VS and NAcc remains an open question in human neurosurgical anatomy. In addition to several case reports or small series [(84), *n* = 3 patients; (85), *n* = 1 patient; (86), *n* = 2 patients; (87), *n* = 1 patient], three larger studies investigated NAcc-DBS for severe and refractory OCD. In Kohl et al. (*n* = 18 patients), bilateral NAcc-DBS was assessed with an open-label design: there were on average 50% responders and 16.7% partial responders at 1 year (88). In one double-blind sham-controlled crossover study, 10 patients received unilateral right NAcc-DBS (89): the mean Y-BOCS score decreased significantly by 21.1% after 1 year of stimulation. In Denys et al. (90) (*n* = 16 patients), bilateral NAcc-DBS was initially performed with an open-label design yielding a 46% Y-BOCS score decrease after 8 months of stimulation, followed by a double-blind crossover phase with a 2-week period of active or sham stimulation enabling a significant 25% reduction of the Y-BOCS score. Permanent adverse events were forgetfulness (31.2%) and word-finding problems (18.8%).

Resting-state fMRI scans in 16 OCD patients undergoing NAcc-DBS and displaying subsequent OCD symptoms decrease revealed reduced excessive fronto-striatal connectivity between NAcc and the LPFC and medial PFC as measured by the blood oxygen level-dependent (BOLD) fMRI signal (10). NAcc-DBS also reduced excessive frontal low-frequency oscillations elicited by symptom-provoking events and measured by scalp electroencephalogram (EEG). Such oscillations are known to appear during goal-directed behavior and to be linked to the severity of some OCD symptoms (91). So, NAcc-DBS might decrease a pathologically excessive fronto-striatal connectivity, allowing the processing of behaviorally relevant stimuli.

Targeting NAcc is challenging since it is a rather large nucleus (10.5 × 14.5 × 7 mm) (92) with complex connectivity (93). Targeting the portions of the NAcc with strongest connectivity to lateral and medial PFC might thus be a promising approach to enhance the effects of NAcc-DBS on OCD symptoms (10). But detailed functional connectivity studies for NAcc-DBS remain to be performed to better understand how a personalized targeting and parameter adjustments might be achieved. Additionally, functional parcellation of NAcc using standard BOLD fMRI protocols with 2-mm isotropic spatial resolution could be already at hand (94), while high-field functional MRI could offer soon spatial resolutions at the scale of DBS electrodes contacts (95).

## Subthalamic Nucleus

The anteromedial subthalamic nucleus DBS (amSTN-DBS) is another therapeutic option to treat severe and refractory OCD and has the strongest evidence in the literature. To our knowledge, amSTN-DBS is associated with the best response rates in randomized and controlled trials, with best long-term outcomes (75% full responders and 53% decrease in OCD severity at 3 years post-surgery) and improvement in global functioning, social, and familial disabilities (96).

In 2002, in two patients suffering from Parkinson’s disease and OCD, STN stimulation for motor symptoms of PD was found to improve preexisting OCD symptoms (11). In 2007, a rationale for this observation was provided when the role of STN in integrating emotional and motor aspects of behavior was demonstrated by performing stimulation of subterritories of the STN in PD patients (97). Indeed, the stimulation of the dorsolateral part of the STN recruits mainly motor networks, whereas stimulation of the anteromedial STN recruits limbic and associative circuits. These authors then reported the first double-blind crossover study performed on 16 OCD patients and demonstrated that 3 months of amSTN-DBS (STOC Study) was sufficient to decrease OCD severity by 39% and improve global functioning by 23% (8, 12). Seventy-five percent of patients were found to be responders. A recent meta-analysis found that 44% of patients could be considered responders to STN-DBS (7). Interestingly, it has been shown recently that amSTN-DBS decreases OCD symptoms for up to 3 years, with a 53% decrease in OCD severity and 92% of patients being considered responders at the final assessment (12 patients in total at 3 years) (96). They also report a positive effect on social activities with a significant improvement in social adjustment (SAS-SR) and work, social, and familial disabilities (SDS).

Early-onset patients were found to have fewer OCD symptoms with STN stimulation. Interestingly, the overall improvement was continuous and progressive with time, with a larger effect of amSTN-DBS on OCD symptoms 46 months after surgery (−51.2% compared with baseline) as compared to 16 months (−16.8% at 46 months compared with 16 months) (51) or 3 months (−39.4% compared with baseline) (12). This highlights one of the major advantages of DBS: the possibility of continuously adjusting the stimulation parameters over time in accordance with patients’ condition to obtain optimal therapeutic effects for each individual.

Nevertheless, psychiatric adverse events related to amSTN-DBS highlight the narrowness of its therapeutic window. At 3 years post-surgery, transient episodes of hypomania and impulsivity due to changes in DBS settings were reported in 33% of patients. However, these changes were short-lived and resolved following stimulation adjustments, as previously reported in 5 of 16 OCD patients (31%) with NAcc-DBS (90) and in 6 of 24 OCD patients (25%) with stria terminalis stimulation (98), suggesting that DBS of these limbic structures may lead to the occurrence of these psychiatric signs (97). It is important to note that no cognitive decline, verbal fluency deficit (99), apathy (100), or significant weight gain (101) was observed in amSTN-DBS OCD patients. Finally, the high rate of suicidal attempts (23%) is in agreement with those encountered in the general population of patients with refractory OCD. DBS may not be the cause since these patients display concomitant increase of impulsivity, anxiety, or depression (96).

Using prospectively acquired DWI and tractography reconstructions in six patients, Tyagi et al. (61) showed that the effective site of stimulation within the STN was located at its inferior medial border, which is directly connected to lateral OFC, dorsal anterior cingulate (DACC), and DLPFC. Among these regions, decreased OFC activity has been shown to be linked to OCD improvement in personalized treatment (102). A resting-state study further showed that abnormal connectivity between lateral OFC and caudate nucleus is associated in OCD patients with errors in the extra-dimensional set-shifting stage 8 (EDS) test, which probes cognitive flexibility (103). Moreover, amSTN-DBS was found to decrease glucose metabolism in lateral OFC with 8-fluorodeoxyglucose positron emission tomography (FDG-PET) performed in 10 OCD patients in a resting state (104). DACC and DLPFC’s activity is linked to the severity of OCD: with fMRI, decreased connectivity between DLPFC and putamen was found in OCD patients and associated with impaired goal-directed planning (103). In addition, tracing studies in non-human primate (NHP) found a hyperdirect limbic pathway from OFC, DACC, and DLPFC to amSTN (62). So, it might be possible that amSTN-DBS interrupts OCD symptoms due to aberrant hyperdirect cortical processing of information by promoting behavioral adaptability. Yet, the sour spots of STN-DBS within STN and its vicinity as well as their functional connectivity remain to be established.

Finally, STN-DBS has been shown to dramatically alleviate motor symptoms in patients with Parkinson’s disease and can be currently performed with optimal target localization within the nucleus (105). The majority of functional neurosurgery centers have experience in targeting STN in the context of movement disorders, so that targeting amSTN for OCD is relatively a direct extension of this technique. Since STN is a much smaller target than ALIC or VC/VS, it also appears less challenging to stimulate the appropriate subterritories displaying the optimal profile of connectivity by adjusting the stimulation parameters and volume of tissue activated (106). amSTN also has a well-characterized electrophysiological signature (107, 108) that helps guide appropriate DBS electrode placement.

## Inferior Thalamic Peduncle

The ITP is a bidirectional fiber tract between the OFC and the thalamus which is thought to play a role in selective attention (109, 110). According to PET studies, OCD patients have increased metabolic activity in the OFC, caudate, and thalamus which is positively correlated with the severity of OCD symptoms (111, 112). In addition, medical treatment of OCD is accompanied with decreased metabolism within a cortico-striato-thalamic circuit (113). Jimenez et al. (14) were the first to report a six-patient case series of ITP-DBS for OCD: all patients were responders and a 51% improvement in Y-BOCS scores at 1 year was found (14). Lee et al. (15) later reported a five-patient case series of ITP-DBS for OCD: all patients were responders with 52% improvement in Y-BOCS at 1 year. FDG-PET imaging in two patients revealed a decreased metabolic level in the caudate, the putamen, and the cingulum after 3 months of ITP-DBS, similar to that observed with SSRIs (113). It is worth noting that adjacent structures, anterior to ITP, such as the BNST, may also be activated during ITP stimulation, according to volume of tissue activated analysis (106). Again, as a fiber pathway, ITP might be challenging to target since there is no known somatotopy or electrophysiological signature which might guide the surgical procedure. Functional studies of this DBS target are unavailable. Above all, larger studies and especially randomized multicenter trials are needed to further investigate this target for OCD treatment.

## Towards the Individualization of the OCD-DBS Target

OCD is a neuropsychiatric disorder encompassing diverse core symptomatic dimensions variably expressed within each patient (114). Indeed, symmetry obsessions/compulsions, contamination and cleaning, aggressivity and checking compulsions, sexual and religious obsessions, hoarding obsessions, and compulsions have been observed in OCD patients (115). These core symptomatic dimensions may be underpinned by separable, partially overlapping neurobiological roles that might respond differently to the stimulation of the different targets we have reviewed (116).

**Figure 3:**
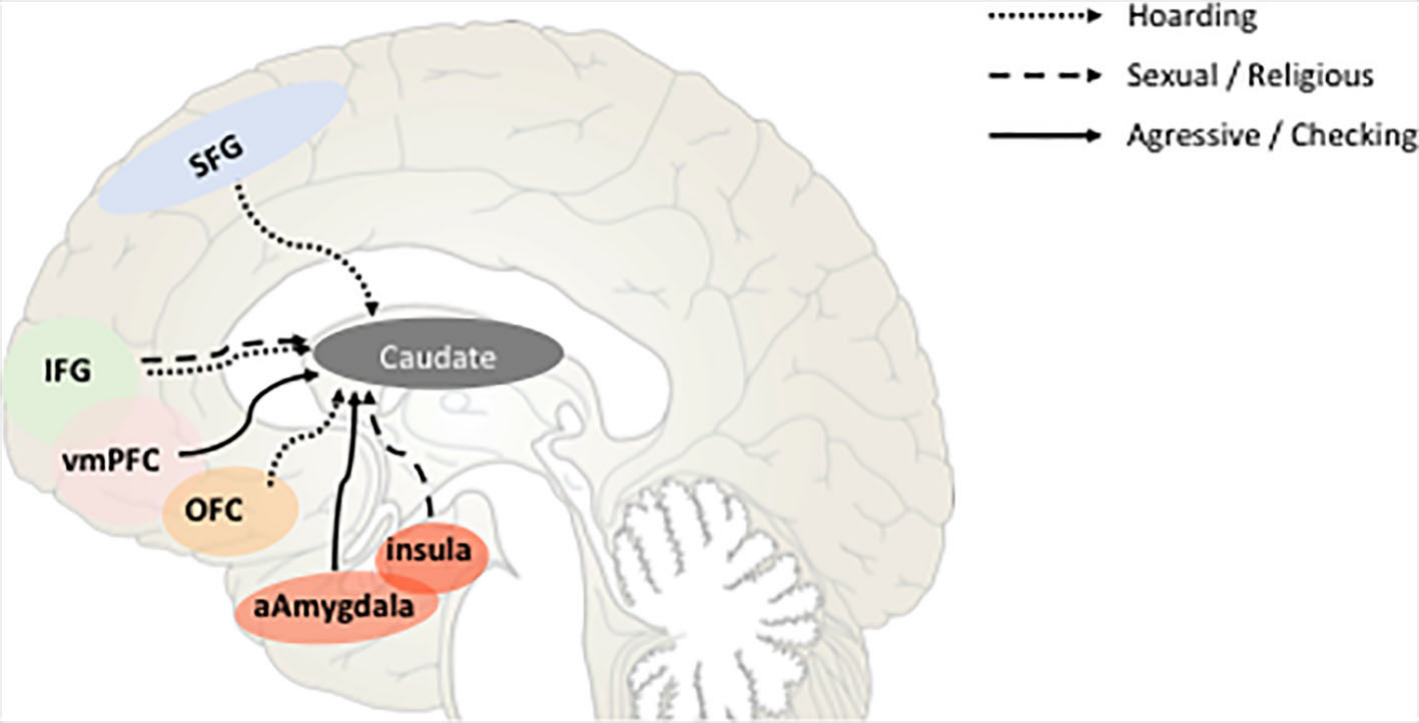
Schematic representation of the correlations between the strength of fronto-caudate connectivity and obsessive-compulsive disorder (OCD) symptoms dimensions according to (26). Sagittal view representing the functional connectivity between different cortical areas known to be involved in OCD physiopathology and the ventral part of the caudate nucleus. Correlations between that connectivity and the different dimensions of OCD symptoms are represented by *full* or *dot lines*. *SFG*, superior frontal gyrus; *IFG*, inferior frontal gyrus; *vmPFC*, ventromedian prefrontal cortex; *OFC*, orbitofrontal cortex; *aAmygdala*, anterior amygdala.

Several recent studies have investigated the functional connectivity of the various DBS targets for OCD in order to define the neural networks to be modulated in order to reach the best clinical response possible. Interestingly, it has been found that the connectivity with the ventral striatum was modulated by different OCD dimensions. Indeed, aggressive/checking symptoms modulate the connectivity of the ventral striatum with anterior amygdala and ventromedian prefrontal cortex (vmPFC), sexual/religious symptoms with insula and inferior frontal gyrus, and hoarding with the OFC (26) (**Figure 3**). Consequently, instead of chasing the “most efficient/best target” dream, one might look for the “most appropriate circuit” for each patient, focusing on their symptom profile or development, age of onset, biological markers, etc.

Barcia et al. (117) pioneered this personalized approach taking into account each patient’s symptom content. They demonstrated that the optimal stimulating electrode contact location for striatal-DBS in each OCD patient is not at a fixed anatomical locus within the ventral striatum or NAcc. The sweet spot lay within different portions of the striatum across patients. In their study, symptom provocation activated specific sites of the PFC. It was already known for example that patients with contamination obsessions mainly activate the medial OFC (ventromedial), while those who present checking symptoms mainly activate the DLPFC (25). They thus showed that stimulating the patient-specific optimal contact location instead of a fixed target increased the response rate to striatal-DBS from 50% (7, 118) to 86%. This concept might also be applicable to other DBS targets for OCD. For example, the ALIC is topographically organized following the same pattern as the fronto-striatal projections (119). The STN has been shown to be segregated into motor, limbic, and associative territories: behavioral functional mapping of STN has been performed in PD patients by stimulation of STN subterritories (97), and anatomical histological tracing of STN connectivity revealed in the NHP connection between the amSTN and the limbic and associative cortical areas as well as between the dorsolateral STN and the motor cortical area (62). It remains to be seen whether neuroimaging and neurostimulation techniques within these STN subterritories will allow us to isolate which networks to activate in order to address specific clinical dimensions based on an individual assessment.

## Conclusion

It appears that the various striatal, thalamic, and STN targets for OCD-DBS that have been under investigation over the past 15 years lie along within same functional neural network. Modulating this thalamo-striatal-frontal network appears crucial in order to improve refractory OCD symptoms. All these DBS targets share similar efficacy profiles when considering improvement of the Y-BOCS score. amSTN-DBS is a therapeutic option for severe and refractory OCD, and the only one with level 1 evidence in the current literature, provided in a randomized controlled trial. Furthermore, amSTN-DBS long-term outcomes are very encouraging, with 75% full responders and 53% decrease in OCD severity at 3 years post-surgery. Randomized double-blind studies comparing targets are not easy to perform, especially when more than two targets are considered.

All things considered, there is room to optimize the choice of targets and of stimulation parameter adjustments for each target under investigation. Choosing the “best” of these targets might require us to take into account at least four points. First, we would like to stress the need for a better understanding of each patient’s specific symptoms and underlying dysfunctional neural networks in order to tailor neuromodulation procedures on an individual basis after symptom provocation procedures and neuroimaging studies for instance. Second, we believe that even though DBS of these various targets might recruit a common network, DBS of each of those targets might also activate further distinct networks responsible for other behavioral effects: amSTN-DBS improves cognitive flexibility, while VC/VS-DBS improves mood and ALIC-DBS might promote flexibility and/or mood (80). Individual characteristics might be crucial when choosing the most appropriate target. Third, surgical difficulty should be taken into account when identifying the optimal target. The STN target is very attractive for DBS since it is a small well-known nucleus with widespread connectivity with the prefrontal cortex. STN-DBS enables modulation of various neural networks that could correspond to various aspects of OCD pathology by simply adjusting the stimulation parameters in order to shape the appropriate volume of tissue activated. For amSTN-DBS, it might thus not be absolutely necessary to perform a preoperative functional connectivity study in order to guide DBS electrode implantation and/or stimulation parameter adjustments, whereas it is more crucial for larger targets such as ALIC, VC/VS, or NAcc. New generations of DBS electrode assemblies which enable steering of the delivered electrical field in order to target therapeutic circuits might thus be valuable for STN-DBS for OCD while at the same time minimizing the stimulation of those circuits responsible for side effects (120). Indeed, sophisticated individualized connectivity studies might not be possible for clinical routine in all DBS centers.

Last but not least, the question of the stimulation frequency and pattern within the various targets mentioned is a question that has not yet been addressed in literature since all targets have been stimulated at high frequency: understanding whether one expects to induce functional inhibition, synaptic plasticity, and specific brain oscillations anterogradely or retrogradely is of the utmost importance (121).

In the future, research efforts should focus on determining the functional connectivity biomarkers to identify best responders and best patterns of stimulation on an individual basis.

## Author Contributions

SS and A-HC conducted the literature search and drafted the manuscript. SP, JY, PD, and LM reviewed and revised the manuscript. JY, SS, and A-HC prepared the figures.

## Conflict of Interest

The authors declare that the research was conducted in the absence of any commercial or financial relationships that could be construed as a potential conflict of interest.
